# Supplementary material for: Patterns of Intron Gain and Loss in Fungi
Source: PLoS Biol. 2004 Nov 30;2(12):e422. doi: 10.1371/journal.pbio.0020422 (PMC532390; doi:10.1371/journal.pbio.0020422)
Supplement: Table S1 — Also available at http://genes.mit.edu/NielsenEtAl/. (4.3 MB ZIP). [file pbio.0020422.st001.zip › NielsenEtAl/html/609.html]

AN0767.1.NCU01930.1.MG02938.1.FG08929.1


```
 CLUSTAL W (1.82) Multiple Sequence Alignments - Introns Inserted


Sequence 1: MG02938.1	146 aa
Sequence 2: FG08929.1	131 aa
Sequence 3: AN0767.1	136 aa
Sequence 4: NCU01930.1	132 aa
Alignment Length: 149 aa
Number Identitical Residues: 72 aa
Alignment Score (without introns) 3069


MG02938.1 	MADRP-HRGGGGGGGNRGNYRGRGGGDRGGRGGRGGDHHHGGKNN-DQQGER-ERPKKEN
NCU01930.1	MADRG-NRGGRGG-----GYRGGRGGNRGGAGGSGSGAGGGQGGGGAQGGDRGERPKKEN
FG08929.1 	MSDRPSHRGG----------RGQGSHPRGGRGGGRG----GGAGG-AQQ-ER-ERPKKEN
AN0767.1  	MSERGSFRGGG-------RNRGGGYDRSGGRGGHGKS---GGAGGGAQQQQQ-EKPKKEN
          	*::* : ***          **      ** **   .   *  ... *  :: *:*****

MG02938.1 	ILDLAKYMDKEVQVKFNGGREV1IGTLKGYDALMNLVLDEVKETMRD1EEGNESSRPLG~
NCU01930.1	ILDLKKYMDQRITVKFNGGREV1TGTLKGYDALMNLVLDDVQEAVR-~----------G1
FG08929.1 	ILDLGKYMDKQITVKFNGGREV1KGTLKGYDALMNLVLDDVHEVVRD1DEGNESTRSLG~
AN0767.1  	ILDLTKYMDKEVRVKFNGGREV1SGILKGYDQLMNLVLDDVKESMRD1DEGNETTRALG~
          	**** ****:.: *********  * ***** *******:*:* :*. .....:: . * 

MG02938.1 	LVVVRGTLLVAISPTDGSESIANPFLQQGDDE
NCU01930.1	LVVARGTLLVVISPVDGSEVIANPFAQQEEEA
FG08929.1 	LVVVRGTLLVLVSPVDGSEEIANPFVQPEDD-
AN0767.1  	LIVARGTLIVLISPADGSEQIANPFVQAEE--
          	*:*.****:* :**.**** ***** *  :
```
